# An improved deep learning model for hierarchical classification of protein families

**DOI:** 10.1371/journal.pone.0258625

**Published:** 2021-10-20

**Authors:** Pahalage Dhanushka Sandaruwan, Champi Thusangi Wannige

**Affiliations:** Department of Computer Science, University of Ruhuna, Matara, Sri Lanka; Taipei Medical University, TAIWAN

## Abstract

Although genes carry information, proteins are the main role player in providing all the functionalities of a living organism. Massive amounts of different proteins involve in every function that occurs in a cell. These amino acid sequences can be hierarchically classified into a set of families and subfamilies depending on their evolutionary relatedness and similarities in their structure or function. Protein characterization to identify protein structure and function is done accurately using laboratory experiments. With the rapidly increasing huge amount of novel protein sequences, these experiments have become difficult to carry out since they are expensive, time-consuming, and laborious. Therefore, many computational classification methods are introduced to classify proteins and predict their functional properties. With the progress of the performance of the computational techniques, deep learning plays a key role in many areas. Novel deep learning models such as DeepFam, ProtCNN have been presented to classify proteins into their families recently. However, these deep learning models have been used to carry out the non-hierarchical classification of proteins. In this research, we propose a deep learning neural network model named DeepHiFam with high accuracy to classify proteins hierarchically into different levels simultaneously. The model achieved an accuracy of 98.38% for protein family classification and more than 80% accuracy for the classification of protein subfamilies and sub-subfamilies. Further, DeepHiFam performed well in the non-hierarchical classification of protein families and achieved an accuracy of 98.62% and 96.14% for the popular Pfam dataset and COG dataset respectively.

## Introduction

Proteins are the main functional body of living organisms. Inside cells, there is a large number of proteins involved in different unique functionalities such as growth and maintenance, causing biochemical reactions, acting as a messenger, providing structures and protection. Therefore, understanding the functionality of proteins is essential for different fields such as drug designing, and disease identification. According to the main functional roles played by proteins, they can be categorized into different groups such as structural, contractile, transport, enzyme, storage, hormonal, and protection [1]. Since the genome projects and technological advancements, the number of known novel protein sequences grew rapidly. Therefore, there is a massive amount of uncharacterized proteins available in the databases. There are nearly 175 million protein sequences in Uniprot according to statistics in 2020 and the lengths of these polypeptide chains vary from 6 to 37,000 amino acid residues [2].

Although the number of protein sequences grows rapidly, these proteins can be categorized into protein families depending on their evolutionary relatedness, similarities in their structure or function. Accurate classification of proteins is important for functional prediction of a given protein sequence and it also helps to provide a more complete picture of the body functionalities of an organism. Protein databases such as GCPR [3], SCOP [4] classify and store proteins hierarchically as classes, families, and subfamilies. COGs [5], Pfam [6], Uniprot [7] are proteins sequence databases that provide both manually and automatically reviewed proteins using computational methods.

Protein characterization to identify protein structure and function is done accurately using biological experiments such as X-Ray crystallography or nuclear magnetic resonance [8]. It is difficult for these experimental characterizations to deal with the rapidly increasing huge amount of novel protein sequences, as the biological laboratory experiments are expensive, time-consuming, and laborious. Therefore, computational methods have been used in protein characterization with the development of computer software and hardware performances [9].

Machine learning methods are widely applied for protein classification. Among the other protein classification methods, Naïve Bayes is a probabilistic classifier based on Bayes theorem used for protein classification. A Naïve Bayes classifier with feature selection was used to classify Phage Virion proteins (Phage is a virus that uses bacteria as hosts) using a benchmark dataset of 307 sequences. This benchmark set includes 99 phage virion protein sequences and 208 Phage non-virion protein sequences from the UniProt database [10]. This method has achieved 79.6% accuracy using the jackknife testing method than the other famous traditional methods used to compare the performance such as Random Forest and Support vector machine. This research on protein classification has also used the Naïve Bayesian tree for multi-level G-protein-coupled receptor(GPCR] classification [3]. Their results show higher accuracy when compared with the other classification methods. However, Naïve Bayes assumes that all attributes are mutually independent and equal in the dataset and this method would also suffer from oversensitivity if there are redundant or irrelevant attributes in the data set.

Random Forest method also has been used for protein fold recognition [11]. Authors have claimed that the Random Forest with a hundred decision trees performed well compared with 17 other different methods. The performance of their method achieved 84.5%, 63.4%, and 40.8% accuracy at protein family, superfamily, and fold levels respectively. However, the Random forest is not much efficient in the classification of temporal data such as sequences and texts as it uses a large number of decision trees. Further, the computational and time costs of this random forest method are high since it generates a large number of decision trees.

Profile Hidden Markov model (pHMM) based protein classification has performed well eliminating the major challenges of alignment-based modeling such as dealing with insertion and deletions of amino acids [12]. pHMM uses a position-specific scoring system according to the level of conservation of each amino acid in each column of the alignment. Profile hidden Markov models (pHMMs) are often used to represent protein families [13]. pHMM models determine homologies among multiple distant protein sequences. As pHMM is one of the state art of modeling methods for protein modeling, a method based on pHMM has also been used in the protein family database, Pfam for protein annotations [6]. In a research-based on profile Hidden Markov Models, authors [14] have built 62 pHMMs to predict and classify the protein type called Cono-peptide superfamilies and families, using various parts of the protein sequences. This model has shown the accuracy of pHMMs as 100% for the classification of the pro and signal-peptides and as 96% accuracy for the mature peptides classification.

With the rapid improvement of the computational performances, deep learning methods have provided better results when compared with the other existing computational methods. Recently many research works have been done using deep learning for protein classification [15,16]. Deep learning methods have shown better performance when it is compared with the existing methods like famous pHMM [17,18] and Random Forest [19]. DeepSF [20] is a 30-layer convolutional neural network (CNN) that classifies protein sequences from 1195 fold classes taken from the SCOP database. This model claims better fold recognition abilities than the state of the art tool HHsearch [21]. However, these deep learning models have not performed so well on finer-grained superfamily and family levels [22].

A model called Deepre [23] combines a CNN with a recurrent neural network (RNNs) and has been used to extract convolutional and sequential features from protein sequences. This was used to predict enzyme classifications. The model was tested using cross-fold validation and the experiments were conducted on two large-scale datasets of the SwissProt database [24].

In another recent work, A deep CNN [25] has also been trained to classify 521,527 sequences from the Uniprot database with 698 families of class labels showing AUC accuracy of 99.99% [26]. It consists of 6 convolutional layers with 2 fully connected layers with nearly 1 million total network parameters. However, deeper neural networks with more parameters give higher accuracy while leading to the overfitting if the number of parameters is too high. ProtCNN [17] (a single deep convolutional neural network), is a recent approach that uses residual networks [27] based deep convolutional architecture to classify protein sequences of Pfam [6] full dataset. In this work, they have compared the performance of ProtCNN with profile HMMs and BLASTp [28] on the benchmark dataset of the Pfam seed dataset. Their model outperformed BLASTp with 200 times faster than BLAST by training 80% of Pfam [6] dataset and also had a lesser error.

DeepFam [18] is a deep learning model that is used for classifying proteins to their families. This model consists of a convolution layer and a fully connected layer. The convolutional layer consists of 8 parallel convolutional units with filter sizes from 8 to 36. This method models arbitrary protein subsequences in a position-specific manner and it provides an end to end model which extracts features and predicts simultaneously. This model is trained and tested using the COG dataset [5] and the cross-validation shows higher accuracy. The model is further used to classify G-protein coupled receptor datasets [3] into multiple levels, with higher accuracy than the state of art methods such as DeepFam and pHMM. However, all of the above discussed models except DeepFam has been tested on non-hierarchical datasets. DeepFam model classifies protein sequences to in multiple levels: families, subfamilies, sub-subfamilies in separate rounds and authors have also emphasized the importance of hierarchal classification or multi-task algorithm in deep learning.

As discussed above, although many methods have been proposed for protein family classification with good accuracies, there is a requirement for a model for the hierarchical classification of proteins at multiple levels simultaneously with high accuracy at a low computational cost. In this research work, we propose a model for the hierarchical classification of proteins at multiple levels based on DeepFam [6] with lesser complexity, computational cost, and training time. We name our model as DeepHiFam and we use the concepts that are used in natural language processing and computer vision for designing the model to achieve better performance. DeepHiFam outperforms other existing models such as DeepFam [18], pHMM, 3-mer logistic regression, Naïve Bayes [3] with the highest accuracies for classes, subfamily, and sub-sub family predicting simultaneously. Further, this model outperforms DeepFam, BiLSTM model, and ProtCNN.

The rest of this paper is organized as follows. Section 2 discusses the methodology including the architecture of DeepHiFam, the model we propose. Section 3 provides the results and discussion of this model. In Section 4, we present the conclusion of this research.

## Methodology

In this section, we explicitly present our improved deep learning model for protein family classification. We conducted our experiment by following several steps that conform to Chou’s 5-steps rule [24]. Since it was proposed in 2011, the “5-steps rule” or “5-step rules” has been widely used in bioinformatics for both theoretical and experimental types of research. The essence of the Chou’s 5 steps is as follows, 1) select or construct a valid benchmark dataset to train and test the model; 2) represent the data samples by encoding them with an effective formulation; 3) introduce or develop a powerful algorithm for predictions; 4) perform cross-validation tests to evaluate the expected prediction accuracy; 5) establish a user-friendly web-server for the model which can be accessed by the public. The following notable merits are achieved by the predictors established in compliance with above-mentioned steps: a) crystal clear in logic development; b) completely transparent in operation; c) easiness to repeat the reported results by other investigators; d) with high potential in stimulating other predictors; e) very convenient to be used by the majority of experimental scientists. Many recent Bioinformatics researches that present predictors using Machine learning methods have used these 5 steps and achieved the advantages of it [29–32]. Accordingly, we have followed this method in our research work and developed a novel deeper model with higher accuracy based on DeepFam [18].

### Benchmark dataset

We have selected the most widely used datasets, Cluster of orthologues (COG) [5,33], and G-protein coupled receptor (GPCR) [3] for testing our model performance that has also been used to train and test DeepFam [18]. COG database presents a phylogenetic classification of proteins from complete genomes and its main purpose is to serve as a platform for functional annotation of newly sequenced genomes [5]. Since 1997, the COG dataset has been publicly available and it includes manually curated protein clusters which provide us an assurance of reliability. GPCR is the largest family of receptor proteins and plays a major role by causing communication among the recognition of different kinds of diverse ligands, including bioactive peptides, amines, nucleosides, and lipids [34].

As shown in Table 1, 6 datasets were used from the selected 3 databases for developing and extensive testing of the proposed model. These datasets helped us for a better comparison of our model with other methods. Our first dataset extracted from the COG dataset includes 1652408 sequences from 4655 families. This number corresponds after filtering the sequences which have a length longer than 1000, as most of the sequence lengths are less than 1000. Further, we filtered the families which have less than 100 sequences since a more balanced dataset always tends to provide the reliability of the measurement of the model accuracy. The final COG dataset is divided into 3 parts that have 100, 250, and 500 sequences per each family (see the first row of Table 1). We name these datasets as COG—A of 1,565,976 protein sequences for 2892 families, COG—B of 1,389,595 protein sequences for 1796 families, and COG—C of 1,129,428 sequences of 1074 protein families respectively. We also use the GPCR dataset [3] which includes 8222 protein sequences for 5 classes, 38 subfamilies, and 86 sub-subfamilies for K-fold cross-validation. Further, we used the GPCR dataset (see the second row of Table 1) to evaluate the model performance in predicting the multiple levels of protein hierarchical classification simultaneously. For further evaluation of our model performance over the methods that have been experimented using the Pfam dataset [6], due to the limited computational power we had, 3000 and 1000 families with respective maximum sequence lengths of 100 and 200 were used to test the model performance (see the third row of Table 1).

**Table 1:** Details of datasets.

DataSet Resource	Dataset Name	Number of Families			Number of Sequences		Sequences Per Family (minimum)
COGs database	*COG-A	2892			1565376		100
	*COG-B	1796			1389595		250
	*COG-C	1074			1129428		500
The BIASPROFS project—GDS	*GPCR	**Classes**	**Sub-Family**	**Sub-SubFamily**	**Traning(80%)/Validation(10%)**		
		5	38	86	8222		10
Pfam database	Pfam seed random split				**Training(80%)**	**Validation(10%)/Test(10%)**	
		1000			439493	54378	200
		3000			715645	87755	98

### Encoding

Humans are capable of working with categorical data directly as their brains can do feature extraction and classification very fast with memorization. For deep learning networks, data sets are needed to be converted into a network understandable format without losing information on the original format. In this research work, we use a similar approach to ordered one-hot encoding [17,18] for converting an aligned protein sequence to numerical vectors. We used 4 steps in encoding a raw sequence to the model input.

**Fig 1:**
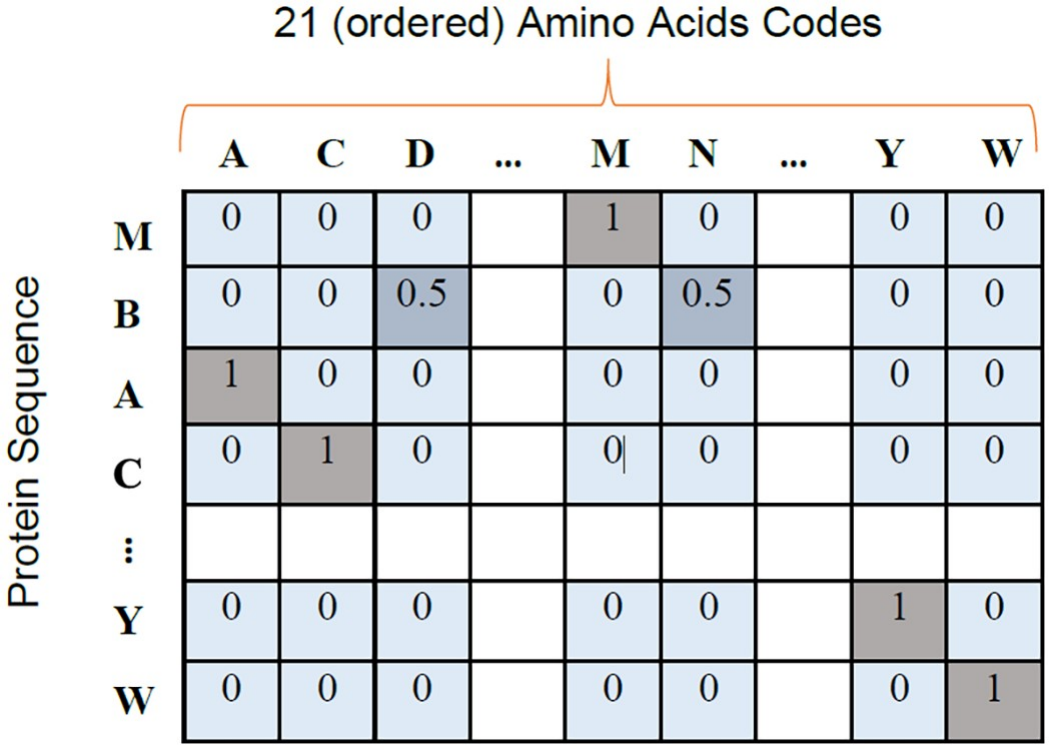
A sequence as a matrix. Representation of a sequence as a matrix (2D array) after encoding the raw amino acid sequence. The x-axis represents invariant 20 positions of amino acids and 1 position for non-amino acids. y-axis represents the sequence with fixed length of at most 1000 amino acids positions.

STEP 1: Padding the amino acid sequence—In the first step, we prepare the sequence length into a fixed value: 1000 to input to the network since each protein has different lengths which were mostly less than 1000 [35]. For instance, when there is an amino acid sequence with 650 amino acids, it is converted as its length into 1000 by appending any special character such as underscore “_” which is not an amino acid.
STEP 2: Designing an amino acid table with numeral codes: We use the IUPAC amino acid naming list [36] in this work to provide each amino acid a number orderly.
STEP 3: Representing each amino acid as a vector in 2D space: We take x-axis as the ordered amino acid codes and y-axis as the amino acids in the sequence as shown in Fig 1. We used non-amino acid positions as zeros and amino acid positions as ones with 2 exceptions as mentioned in the next step 4.
STEP 4: Encoding labels: Each label is provided with an integer code and represented using ordered one-hot encoding. For the hierarchical classification of multiple levels, we created 3 label sets per sequence for multi-label classification of the GPCR dataset.

### Architecture

#### The model (DeepHiFam): Multi-scale convolutional neural network

**Fig 2:**
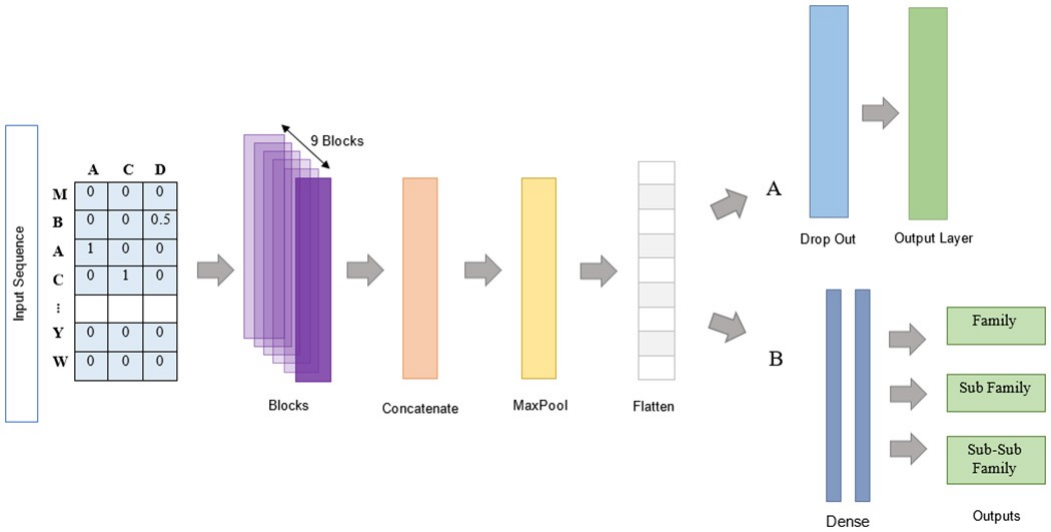
The architecture of the proposed feedforward DeepHiFam model. Encoded sequences are fed into the feature extraction blocks. Extracted feature maps are concatenated before the 1D max pool layer which reduces the dimensionality. After the flattening layers, two different classification sections are separately designed as shown in parts A and B for non-hierarchical and hierarchical multi-class classification respectively. The classification section-A has a fully connected (Dense) layer with softmax activation function. Section-B has multi-outputs which include fully connected layers that are connected to the final fully connected layers. Dropout layers are also used with a 0.5 rate after the flattening layer and the fully connected layers to avoid the overfitting of the model.

We designed the model as a multi-scale convolutional neural network. Fig 2 summarizes the details of the designed model. The model consists of 2 main sections. Those are the feature extraction section and the classification section. The classification section is further divided into two sections with a single output (Fig 2A) and multiple outputs (Fig 2B). The feature extraction section consists of an input layer, blocks of feature extraction units, the concatenation layers, max-pooling layer, and flattening layer. The blocks of feature extraction are designed using the concept of residual blocks of the ResNet [27].

**Fig 3:**
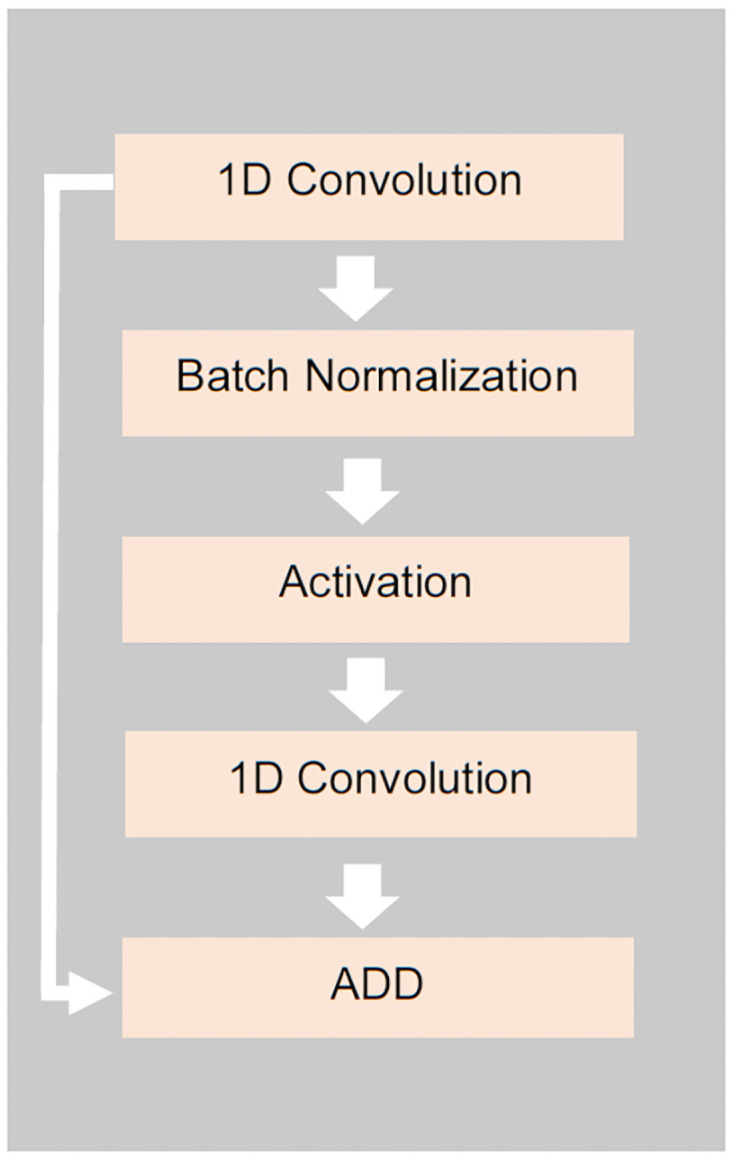
The architecture of a block of the feature extraction section of the proposed DeepHiFam. First, encoded sequence is fed into the 1st convolution unit. The extracted feature maps are batch normalized and input to the Relu activation, which helps to improve the speed, performance, and stability. The output of the 2nd convolutional unit is added to the output of the 1st convolution unit. 9 parallel blocks of different kernel sizes (see Table 3) are used for feature extraction.

We re-designed the model network adding a block of the feature extraction as shown in Figs 2 and 3. The single block of the blocks used in the feature extraction unit (Fig 3) consists of a sequentially connected convolution unit, batch normalization unit, activation unit, and another convolution unit respectively. The first convolutional layers of all blocks are parallelly connected to the input layer. We can consider the 1^st^ level convolutional layers of all blocks as a set of 9 parallel convolution units. In the 1^st^ level, multi-scale kernel sizes are from 8 to 40. Each output feature map of each 1^st^ level convolutional layer is batch normalized to standardize the output of the 1^st^ level to input to the next level and then activated to introduce the non-linear property to the output using the rectified linear unit (ReLU) activation function. After that, all the feature maps are concatenated and the max-pooling layer is used to reduce the dimension. Next, the dimension reduced output of the max-pooling layer is flattened for classification. A drop out layer is also used to avoid over-fitting.

A final fully connected layer with softmax activation is used to facilitate protein multiclass non-hierarchical classification (Fig 2A). For the hierarchical classification of the protein families (Fig 2B), we design the classification section of DeepHiFam with 03 separate output layers of 2 dense layers each, along with a drop out layer to avoid over-fitting. Accordingly, we designed the model as a multi-scale multi-output convolutional neural network with a lesser number of parameters and more generalization compared to the existing deep learning models. All the hyper-parameters of the model are shown in Table 2.

**Table 2:** Hyper-parameters of DeepHiFam.

Parameter	Range
Sequnce Length	1000
Batch size	100
kernel sizes (1st Layer)	[8,12,16,20,24,28,32,36,40]
Number of filters	250
kernel sizes (2st Layer)	4
Activation	Relu
Learining rate	0.0001
Epochs	15
Early stopping patience	2
Loss function	Categorical Cross Entropy
Kernel Resgulizer	L2(0.0001)
Dropout	0.5

The model was trained and tested for single layer output classification using the COG and Pfam datasets. GPCRs hierarchical dataset was used for the classification of protein families. In the classification (Fig 2B), 2 fully connected layers with the sizes of 1000, 500 hidden units, 1 fully connected layer with 500 hidden units, and another 1 fully connected layer with 800 hidden units were used for family, subfamily, and sub-subfamily classification along with a dropout layer. Early stopping method was used which helps to get rid of the problem of choosing of the number of training epochs to use. Early stopping patience as 2 was used for hierarchical protein classification. We paid our attention to maintaining a lesser number of parameters when designing the model. Adding more convolutional units with higher numbers of filters to the model increases the computational cost and the training time significantly. However, we used the “identity shortcut connection” that is used in ResNet [27] architecture to remove the gradient decency error of a deeper network. By using this method, we were able to reduce the number of parameters and, batch normalization and Relu activation were used to improve the model performance, speed, and stability.

In this research, all computational experiments were carried out with google co Laboratory-Pro with P100 GPU support [37]. The model was implemented in python using TensorFlow Keras. Tensorboard was used to visualize the metrics over epochs [38]. All Source codes and Datasets are available at the following github repository. (https://github.com/SanduDS/Research_Protien_Classification).

## Results and discussion

### Evaluation metrics

We used the following evaluation metrics to have a broader understanding of the learning process of the model and to study the performance of the models. We use widely used measures: accuracy, precision, recall, F1-Score, and area under the receiver operating characteristics (AUROC) which are further discussed in the following section.

#### Accuracy

This is an important performance measurement of a model that measures the ratio of correct prediction amount to the total amount of data samples as defined in Eq (1). TP = True Positives, FN = False Negative, TN = True Negatives, FP = False Positive

$$Accuracy=\frac{\left(TP+TN\right)}{\left(TP+TN+FN+FP\right)}$$ (1)

#### Precision

Accuracy may not be enough to understand the complete picture of the model performance if the dataset is imbalanced [37]. Precision provides a measurement of the model performance when the dataset is imbalanced. Further, precision is important when studying the class-wise performance of the model. Precision is defined in Eq (2) by having the ratio of the number of true-positives and the number of true and false-positive predictions of a model.


$$Precision=\frac{\left(TP\right)}{\left(TP+FP\right)}$$ (2)

#### Recall

As defined in Eq (3), Recall is the metric that provides a measurement of the fraction of samples from a class that is correctly predicted by the model [37].


$$Recall=\frac{\left(TP\right)}{\left(TP+FN\right)}$$ (3)

#### F1-score

F1_score shown in Eq (4), provides a measurement to take the overall idea of model performance when both the recall and precision are important. Protein sequence classification is an application area where the attention should be paid not only to the number of correct predictions but also to the overall correct and incorrect prediction of the model [37].


$$F1-score=\frac{2\times Precision\times Recall}{Precision+Recall}$$ (4)

#### AUROC

Area under the ROC is the measurement that shows how the model performs at all classification thresholds. True positive and False positive rates are used to plot the curve. It measures the 2-dimensional area covered by the entire ROC curve. Better AUC usually lies between 0.9 and 1 [38].

### Comparison of DeepHiFam performance with COG dataset

The model is trained using 3 -Fold cross-validation for COG datasets: A, B, and C. As 3-fold cross-validation provides validation of the dataset using multiple folds, it helps to have an idea of the generalizability of the model. One of Tensorflow-Keras input methods for large datasets (Keras.utils.sequence returning inputs and targets) is used as the memory was not enough to completely load full training or test dataset at once. Further, categorical cross-entropy was used as the loss function of the model as our application is a multi-class classification problem. Adam optimizer was used for model optimization.

**Fig 4:**
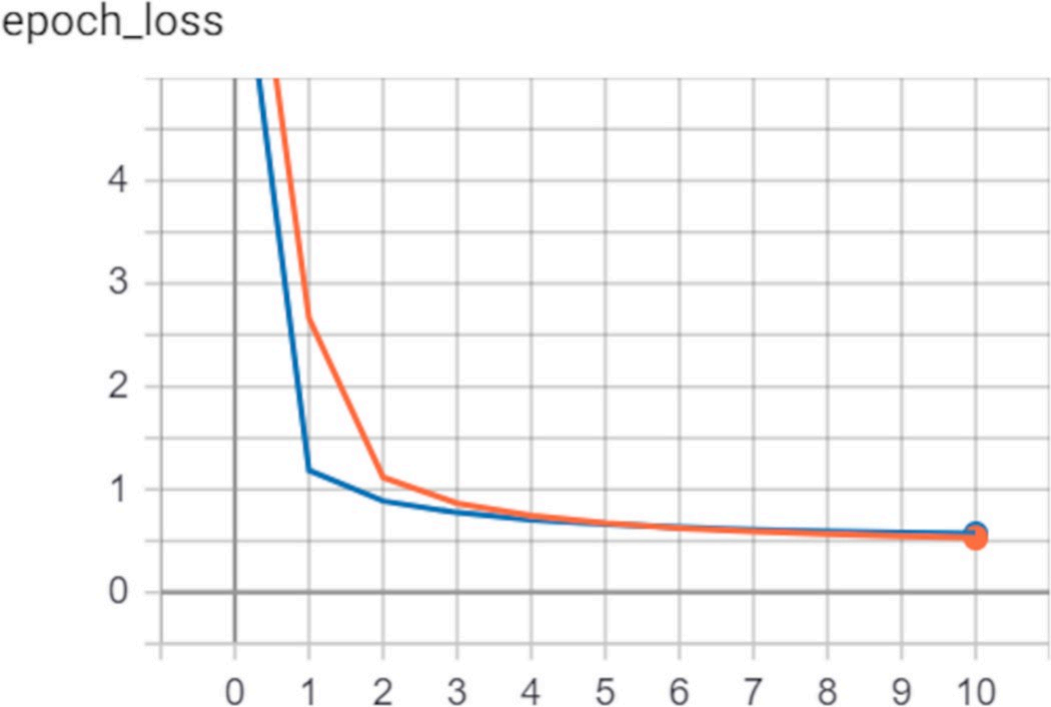
Loss value graph of validation and train at the 1st iteration of the of COG-A dataset. It shows that the DeepHiFam is not overfitting as there is rarely a gap between two curves.

Three datasets COG A, B, and C as mentioned in Table 1 were used. Table 3 shows the results of the application of 3-fold cross-validation to analyze the models. DeepHiFam shows higher accuracy and F1-score which is the weighted average of precision and recall (sensitivity) of the model. This shows that the model performs better not only because of the high accuracy but also considering the false positives and false negative predictions of the model. Fig 4 shows the curves of the losses values of training and validation at the 1^st^ iteration of the COG-A dataset. The loss value graph was randomly chosen as we have obtained all the training and validation graphs of the model. These graphs show no wide gap between training and validation curves. This indicates that the model performed well while using COG dataset.

**Table 3:** 3-fold cross-validation results of COG A, B, and C dataset (see Table 1).

Iteration Number	Number of Classes	Model: DeepHiFam				
		Accuracy(%)	Precision(%)	Recall(%)	AUC(%)	F1-Score(%)
**0**	2892	94.95	97.05	93.14	99.45	95.05
**1**		94.97	97.13	93.15	99.46	95.10
**2**		94.87	96.97	92.81	99.42	94.84
**Average**	COG-A	**94.93**	97.05	93.03	99.44	95.00
**0**	1796	95.32	97.11	93.86	99.55	95.46
**1**		95.65	97.29	94.26	99.59	95.75
**2**		95.62	97.22	94.28	99.54	95.73
**Average**	COG-B	**95.53**	97.21	94.13	99.56	95.65
**0**	1074	96.16	97.46	95.06	99.67	96.25
**1**		96.18	97.51	95.03	99.70	96.25
**2**		96.08	97.45	94.91	99.68	96.16
**Average**	COG-C	**96.14**	97.47	95.00	99.68	96.22

We compared the prediction accuracy of DeepHiFam with other models: DeepFam, pHMM,3-mer Logistic Regression, ProtVec Logistic Regression for protein classification. As shown in Table 4, our model has the highest accuracy compared to the other models that were compared using the COG dataset. Further, DeepHiFam has a lesser number of parameters than the other existing deep learning methods which makes the model a more generalized one with the highest accuracy. DeepHiFam is deeper neural network than the DeepFam model which consists of one convolutional layer with different filter sizes.

**Table 4:** The prediction accuracy (%) comparison of COG A, B and C datasets.

Dataset	COG-A	COG-B	COG-C
The proposed model	**94.93**	**95.53**	**96.14**
DeepFam*	91.40	94.08	95.40
pHMM*	91.67	91.78	91.75
3-mer LR*	75.44	81.15	85.59
Protvec LR*	37.05	41.76	47.34

Bold values are the highest/* Results obtained from DeepFam [18].

### Comparison of the model performance with Pfam dataset

A protein family dataset from the Pfam database was also used to compare the model performance with the other deep learning methods which has shown higher accuracy on classification: BiLSTM network [39] and ProtCNN [17] based classification model. We tested the DeepHiFam model with two different sequence lengths and the number of classes 250, 1000, and 100, 3000 respectively as shown in Table 5. The results in Table 5 show that DeepHiFam has the least number of parameters than the other two considered networks. Further, it shows the highest accuracy compared with the other models.

**Table 5:** Parameter comparison with ProtCNN and bi-directional LSTM.

Method	Number of Class	Accuracy(%)	Number of Parameters
The Proposed Model	1000	**98.94**	9,611,000
	3000	**98.04**	10,613,000
ProtCNN	1000	98.74	10,761,448
	3000	97.55	12,811,448
BI. LSTM	1000	96.82	231,016
	3000	95.66	489,016

**Fig 5:**
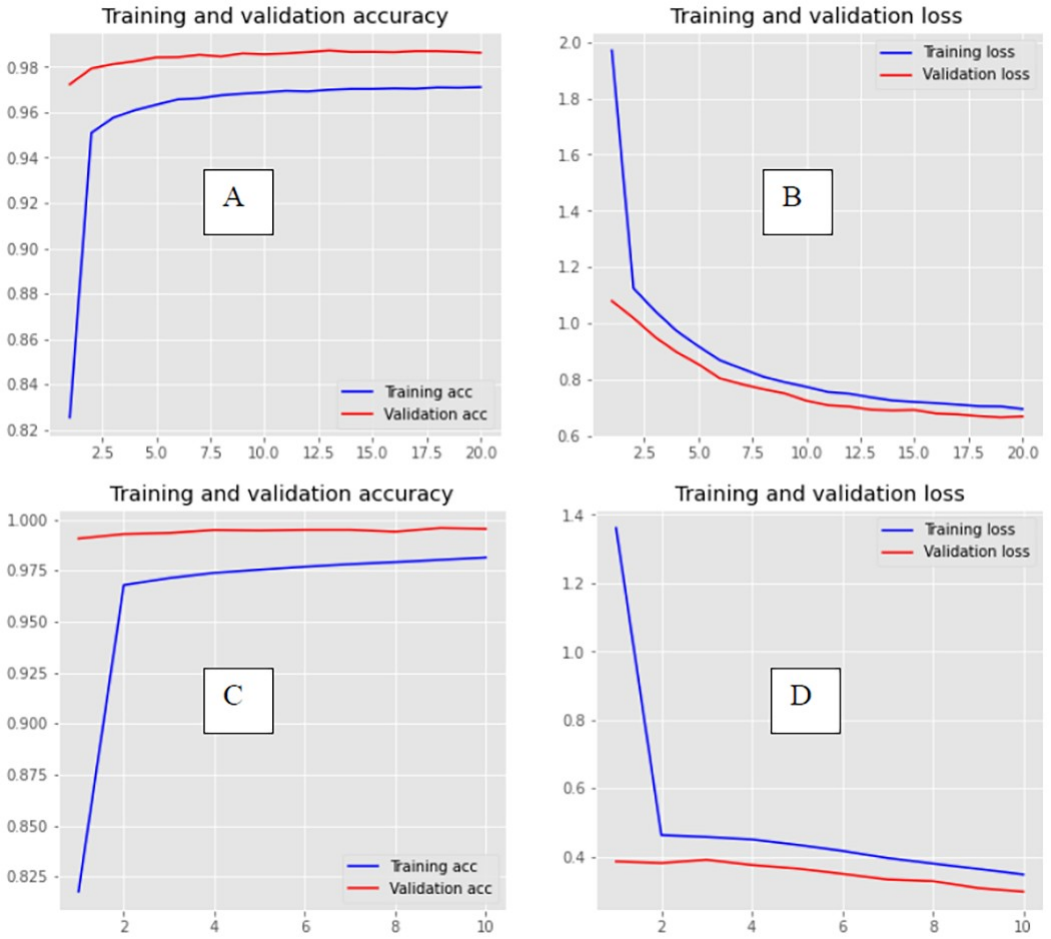
Training and validation accuracies without overfitting. (A, B- 1000 classes and 250 sequence length, C, D- 3000 classes and 100 sequence length) Left: Shows DeepHiFam model’s training and validation accuracy vs number of epochs. Right: Shows DeepHiFam model’s loss vs number of epochs. Both graphs show how the model learns without over-fitting.

The graphs of loss values of training and testing of DeepHiFam model show how it reached the highest accuracy without overfitting (Fig 5). This is mainly because the training loss values of every epoch are lesser than the testing loss values. However, after some epochs, ProtCNN tends to have lesser loss values of training than testing. It also shows the losses and accuracy graphs of the model highlighting the difference between training and testing approaches of the model for different sizes of outputs. It gives a clear idea of how well this model performs by presenting the training and testing behavior of the deep learning model. This concludes that DeepHiFam model has better performance and generalization than the other existing methods in protein classification.

### Hierarchical protein classification of the model using -G Protein-Coupled Receptor (GPCR)

The GPCR dataset (see Table 1) is used to evaluate the model performance in predicting the multiple levels of protein hierarchical classification simultaneously. In other available models such as DeepFam [3,18] the classification of the proteins is done separately in separate rounds.

We used 10-fold cross validation [40] for hierarchical classification. The full data set with 7938 protein sequences belonging to 5 families, 38 subfamilies and 86 sub-subfamilies was randomly split into ten disjoint subsets, each containing (approximately)10% of the data. The model was trained on the training set and then the validation was carried out. We have used the same dataset and the approach when using the dataset according to the DeepFam [18] to ensure a better comparison and fair experiment. Categorical cross-entropy was used as the loss function and Adam optimizer was used which optimizes the model performance by updating the weight parameters to reduce the loss.

**Fig 6:**
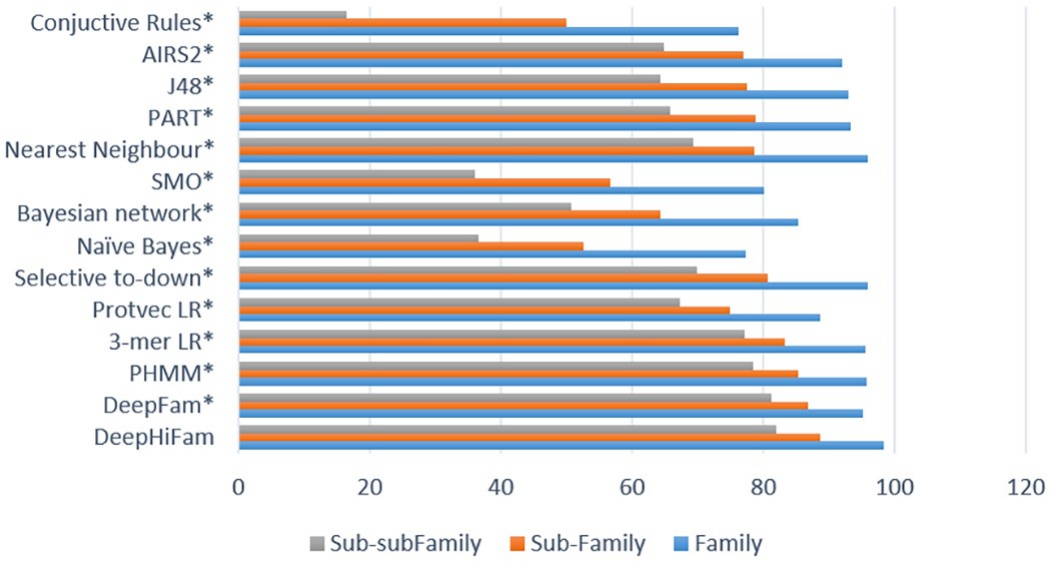
Prediction accuracy (%) comparisons of GPCR dataset. Results are extended from DeepFam [18]. This chart shows that our model has the power of hierarchical classification simultaneously using multi-outputs.

Fig 6 shows the accuracy comparison of different types of other existing methods. DeepHiFam performed well in the hierarchical classification. The model achieved the highest accuracy with parallel multiple outputs for 3 different levels and a lesser number of parameters. These results show that this model can be used to hierarchically classify protein sequences into correct levels simultaneously. Furthermore, (See Table 6) when the AUC value of each experiment is considered, it always varies between 93% and 100%, indicating that DeepHiFam model can perform well in distinguishing between classes. There is a high accuracy in the proposed model of more than 1% from DeepFam model in Family level classification while other models achieved lesser accuracy around 95% or less. In the next hierarchical level, proposed model has shown the highest accuracy than the other models with a 2% difference than the 2nd better performer at subfamily level. It clearly shows that our model has higher accuracy as a hierarchical classification network. In the sub-sub family level, most of the models are achieving accuracy around 60% and 50%. However, our model has achieved 82.05% average accuracy than every method we compared.

**Table 6:** Parameter comparison with ProtCNN and bi-directional LSTM.

Method	Family	Sub-Family	Sub-subFamily
The Proposed Model (DeepHiFam)—from single round of running	**98.38**	**88.69**	**82.05**
DeepFam*	97.17	86.82	81.17
PHMM*	95.77	85.39	78.5
3-mer LR*	95.59	83.39	77.06
Protvec LR*	88.58	74.98	67.32
Selective to-down*	95.87	80.77	69.98
Naïve Bayes*	77.29	52.60	36.66
Bayesian network*	85.24	64.27	50.69
SMO*	80.21	56.67	35.96
Nearest Neighbour*	95.87	78.68	69.40
PART*	93.27	78.73	65.68
J48*	92.93	77.49	64.30
AIRS2*	91.98	76.92	64.78
Conjuctive Rules*	76.19	49.93	16.49

*Bold values are the highest./*-Results from DeepFam [18].

Not only our model has performed better than the other model which had been tested using the same dataset but also our model performed well at one time prediction for all three families rather than running the model for sevel time for each family level as most of the methods predict multiple levels separately in several rounds.

## Conclusion

Protein family classification is very important in protein function prediction, drug designing, and in disease discovery. In protein family classification, deep learning-based models have achieved higher accuracy. Most of the currently available deep learning models in protein classification carry out the classification of proteins in a single layer of the protein hierarchy. DeepFam, which is a deep learning model introduced recently, is used for hierarchical classification separately in different rounds. Here, we propose a deep learning neural network model called DeepHiFam with higher accuracy and a lesser number of parameters to classify proteins hierarchically into different levels simultaneously. DeepHifam also shows high accuracy in the non-hierarchical classification of proteins, outperforming the available models.

DeepHiFam achieved higher accuracies in classifying COG and Pfam datasets than the existing methods we compared. Not only that, DeepHiFam model performed well in hierarchical protein classification of the GPCR dataset using multiple output layers with the highest accuracy than DeepFam and other existing models. The proposed architecture can be applied generally to any hierarchical classification or classification problem.
